# The alteration of gut microbiome and metabolism in amyotrophic lateral sclerosis patients

**DOI:** 10.1038/s41598-020-69845-8

**Published:** 2020-08-03

**Authors:** Qianqian Zeng, Jie Shen, Kangzhi Chen, Jinxia Zhou, Qiao Liao, Ke Lu, Jiao Yuan, Fangfang Bi

**Affiliations:** 1 Department of Neurology, Xiangya Hospital, Central South University, No. 87 Xiangya Road, Kaifu District, Changsha City, 410008 Hunan Province China; 2 Department of Neurology, The First Traditional Chinese Medicine Hospital, Changde, 415000 China; 3 Eight-Year Program of Clinical Medicine, Xiangya School of Medicine, Central South University, Changsha, 410013 China

**Keywords:** Neuroscience, Diseases, Neurology, Pathogenesis, Risk factors

## Abstract

Amyotrophic lateral sclerosis (ALS) is a neurodegenerative disease accompanied with severe paralysis or even death, while the pathogenesis of ALS is still unclear and no effective therapy exists. The accumulating evidence has indicated the association between gut microbiota and various neurological diseases. Thus, to explore the potential role of gut microbiome in ALS, 20 patients diagnosed with probable or definite ALS and 20 healthy controls were enrolled and their fecal excrements were collected. The analysis of fecal community diversity with 16S rDNA sequencing showed an obvious change in microbial structure of ALS patients, where Bacteroidetes at the phylum level and several microbes at the genus level were up-regulated, while Firmicutes at the phylum level and *Megamonas* at the genus level were down-regulated compared to healthy controls. Additionally, decreased gene function associated with metabolic pathways was observed in ALS patients. The metagenomics further demonstrated the discrepancies in microflora at the species level and relevant metabolites thereof were also revealed when combined with metabolomics. In conclusion, the altered composition of the gut microbiota and metabolic products in ALS patients provided deeper insights into the pathogenesis of ALS, and these biomarkers might be established as potential therapeutic targets which deserve further exploration.

## Introduction

Amyotrophic lateral sclerosis (ALS), a progressive and ultimately fatal motor neuron disorder with both brain and spinal cord involvement, is generally characterized by limb or bulbar related symptoms as initial clinical manifestation whereas respiratory compromise the end-stage presentation^1^. The average survival duration of the sufferers is reported to be 3–5 years from ALS onset, while the prognosis depends on multiple factors including disease phenotype and varies with the age, gender as well as the nutritious condition of the patients^1–3^. Most cases fall within the category of sporadic amyotrophic lateral sclerosis and around 5–10% of patients exhibit a gene-related trend. Mutations of *SOD1*, *FUS*, *C9orf72*, and *TARDBP* are more commonly associated with ALS^4^. Thus, together with the role of genetics, partially interpreted by several hypotheses which involve disturbances in RNA and protein homeostasis as well as energy metabolism notwithstanding^3,4^, the precise pathogenesis of ALS remains to be better elucidated. Additionally, in spite of massive efforts having been invested, there is no cure available at present and merely riluzole (glutamate antagonist) and edaravone (free radical scavenger) were approved for ALS treatment by Food and Drug Administration (FDA)^5,6^, which could only slow the development of the disease to some extent^7,8^. Hence, it is imperative that the underlying nosogenesis of ALS be unraveled and effective therapy be carried out to minimize the sufferings of patients.

As the most abundant microorganism being interdependent with human hosts, the gut microbiota not only constitutes a specialized microecosystem in gastrointestinal (GI) tract and harmonizes their relations dynamically, but is engaged in metabolic regulation and immune defense through the interplay with multiple distant organs and systems beyond the intestine^9^. In recent years, extensive studies have revealed that the microbiota–gut–brain axis is closely implicated in the pathophysiology of neurological diseases, which include but are not limited to Alzheimer’s disease^10^, autism spectrum disorder^11^, stroke^12,13^, multiple sclerosis^14^, and Parkinson’s disease^15,16^. As a crucial part of the axis, intestinal barrier dysfunction played an essential role in both gastrointestinal inflammatory diseases and neuropathology as well^17^. The microglia and astrocytes in central neuronal system were attested to be regulated by metabolites derived from symbiotic gut microbes, the pathway of which inhibited the neuroinflammation and neurodegeneration in the experimental autoimmune encephalomyelitis model^18^.

In a similar manner, substantial evidence existed to support the notion that gut microbiome alongside with small molecule metabolites derived from GI tract played a role in the pathogenesis of ALS when they reached the central nervous system through the blood–brain barrier^19,20^. In the G93A-SOD1 transgenic mice models for ALS, it was shown that the impaired intestinal epithelium and tight junction potentially contributed to the progression of ALS^21^, and that replenishment of probiotics and the relevant metabolites thereof ameliorated the motor ability of mice^21,22^. It was of essential note that the gut microbiota constitution in mice had been altered prior to the development of motor neurons dysfunction, and consequently the dysbacteriosis could act as one of the possible mechanisms for the ALS onset^22^. Nevertheless, there has been some inconsistencies as revealed in other studies. A disequilibrium in intestinal microbiota composition was found after analyzing 6 ALS patients and 5 healthy controls^23^, while no significant alteration was indicated in another work involving 25 ALS patients and 32 healthy individuals^24^. It is widely shared that a multitude of factors can influence the colonization of gut microbiota, including diet, drugs, and stress^25^. In this sense, the relationship between ALS and gut microbiota is probably not overwhelmingly conclusive and dependent on a variety of factors. Thus, in the present study, metagenomics sequencing and metabolomics were applied to determine the discrepancies in gut microflora diversity and associated metabolism between ALS patients and their healthy family caregivers, which provides further evidence to assess the causative role of microbiome in ALS.

## Results

### Characteristics of study population

Twenty ALS patients and an equal number of age-matched healthy controls were recruited, in which each group consisted of 8 females and 12 males. All ALS patients meet the standard of diagnosis according to revised El Escorial criteria, and were diagnosed by 2 experienced clinical neurologists. None of the participants has a history of diseases or other factors that are potentially contributing to the alteration in the gut microbiota. All the enrolled participants were from distinct regions in the Hunan Province of China and there were no racial differences in the population. Demographic information is listed in Table 1 for all subjects/patients.

**Table 1:** Characteristics of study participants.

Parameter	ALS^a^	CON^b^	P value
Number of participants	20	20	0.5
Age (years old)	53.9 ± 9.6	50.6 ± 12.6	0.434
**Gender**			
Female	8	8	0.5
Male	12	12	0.5
BMI (Kg/m^2^)	22.8 ± 2.2	21.8 ± 3.2	0.148
Peripheral neuropathy	0	0	–
Gastrointestinal tract diseases	0	0	–
History of gastrointestinal surgery	0	0	–
Diabetes	2^d^	0	–
Dieting	0	0	–
Hypercholesterolemia treatment	0	0	–
**Type of onset**			
Bulbar	4	–	–
Spinal	16	–	–
Duration(months)	10.7 ± 1.6	–	–
ALSFRS-R score	38.4 ± 1.3	–	–
ALSFRS-R point loss^c^	7.4 ± 1.2	–	–
Bulbar symptoms at the time of specimen collection	13	–	–
Enteral or parenteral nutrition	0	–	–
Non-invasive ventilation	0	–	–

Most of the data is presented as mean ± SD.

^a^Probable or definite clinical diagnosis of ALS patients according to revised El Escorial criteria

^b^Healthy controls

^c^Mean number of ALSFRS-R point loss from disease onset to specimen collection

^d^Neither of the two patients used drugs to control blood glucose nor were on a diet.

### The diversity and taxa of the fecal microbiota

**Figure 1:**
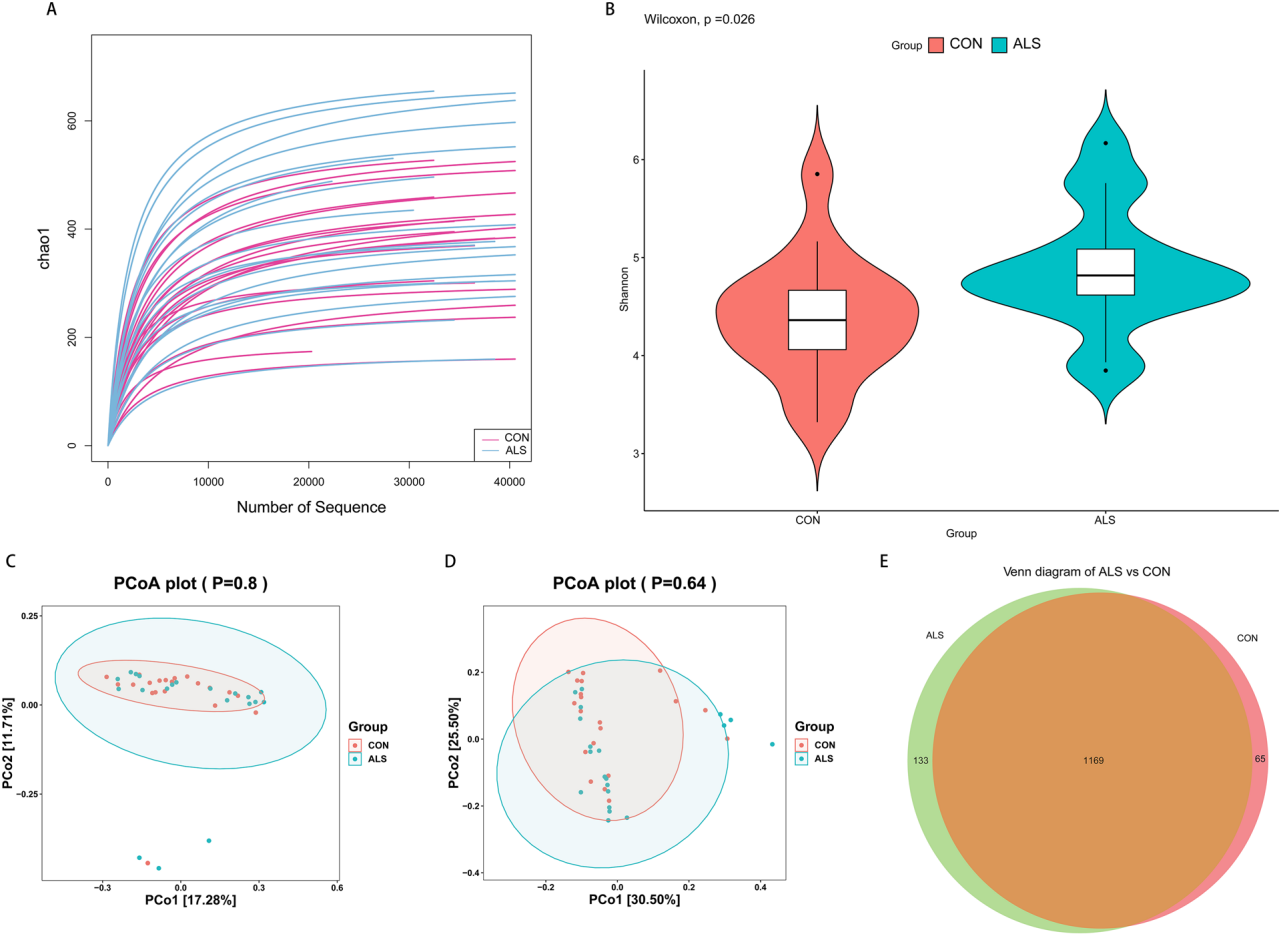
The preliminary comparison on diversity of gut microbiota between the ALS and CON group. (**A**) The quantities and differences of OTUs among the samples are directly shown in the rarefaction curves of Chao1 index, which tend to flatten and suggest the sufficiency and reasonableness of the sequences. (**B**) The boxplot of Shannon index shows the difference in OTUs diversity between the ALS and CON group (p = 0.026). (**C**,**D**) PCoA with unweighted (p = 0.8) and weighted UniFrac distances (p = 0.64) indicate the between-sample discrepancies in microbial structure are not significant. PCo1 and PCo2 are used to plot the coordinate axis and the percentages refer to the extent of variation explained. (**E**) Venn diagram intuitively present the number of the common and exclusive OTUs between the ALS and CON group calculated through the R software.

The rarefaction curve of Chao1 index suggested that the diversity of OTUs in ALS group was relatively more abundant as indicated by the higher curves thereof under the same sequencing depth (Fig. 1A). On the other hand, all the curves tend to be smooth and reach the plateau, which indicates that the amount of sequencing data was sufficient and reasonable, and that more data would only produce a few of new OTUs. In addition, we found a significant difference in microbial richness and evenness between the ALS and controls as demonstrated in the boxplot of Shannon index (P = 0.026, Fig. 1B). To identify the similarity between the samples, we performed PCoA with UniFrac distances. One of those, unweighted UniFrac distance, highlights the differences between samples attributed to the discrepancies in community composition, while the other, weighted UniFrac distance, places more emphasis on the change in abundance gradient of different community species. No significant variations were found between both groups with PCoA (unweighted UniFrac, P = 0.80; weighted UniFrac, P = 0.64, Fig. 1C,D). However, the Venn diagram (Fig. 1E) exhibited a distinction and visually illustrated the exact number of OTUs common (1,196) and unique (133 for ALS and 65 for controls) to the groups. Furthermore, the ANOSIM also showed the structure of the gut microbiota of the ALS group was significantly different from that of the controls (weighted UniFrac, R = 0.08, p = 0.03, Table 2). These data preliminarily clarified that the structure of intestinal microbial community was slightly altered in ALS group.

**Table 2:** Analysis of similarities (− 1 $\le$ R $\le$ 1, P < 0.05).

Method	R statistic	P value
Unweighted UniFrac	0.04	0.10
Weighted UniFrac	0.08	0.03

**Figure 2:**
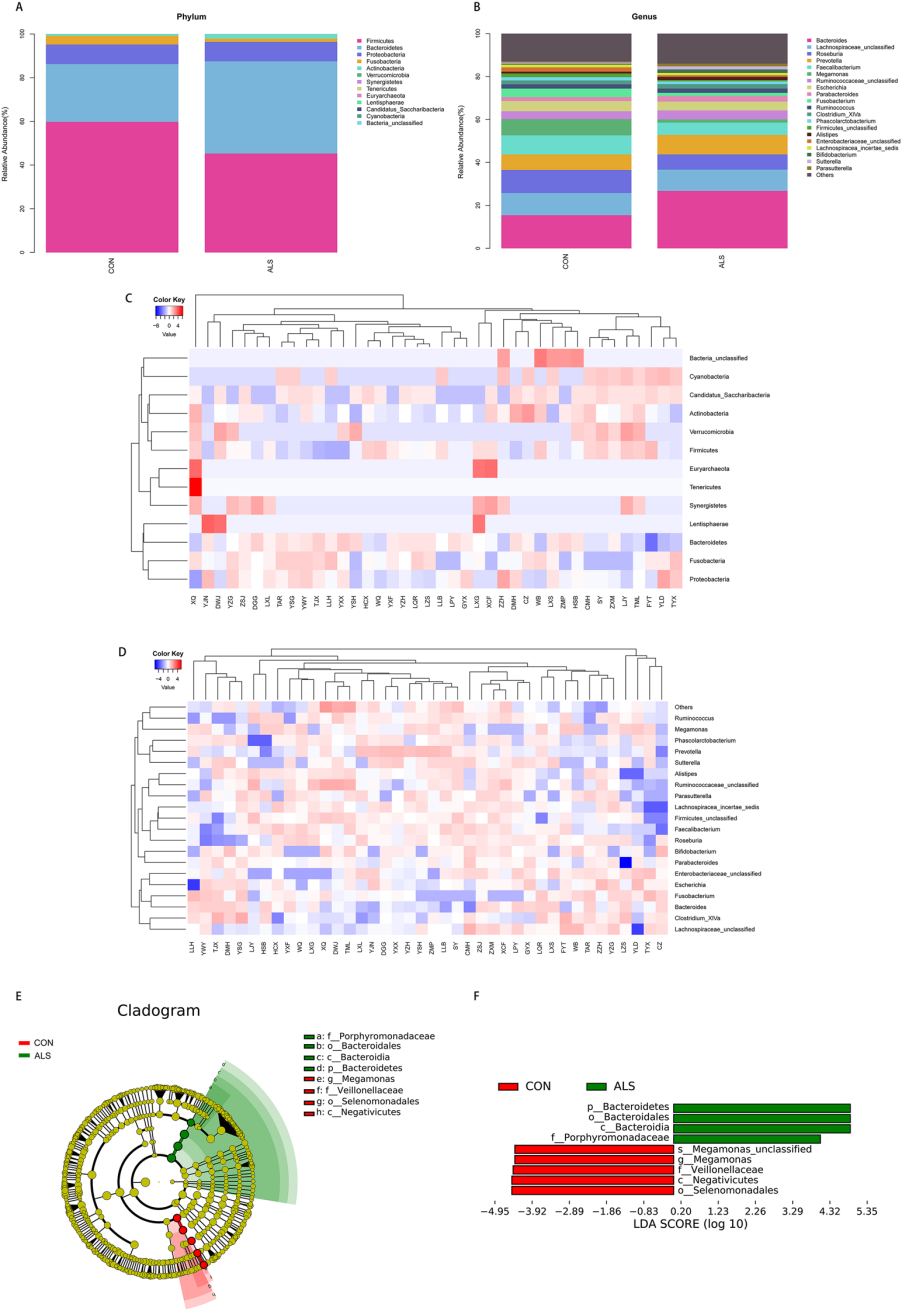
The taxonomic characterization of gut microbes and biomarkers in ALS and CON group. (**A**,**B**) The taxonomic distributions and relative abundance of the fecal microorganisms from both groups are shown in stacked bar charts at the phylum level and the genus level (only top 20 displayed). Different colors represent different microbes at the same level. The bar from bottom to top corresponds to the relative abundance from high to low. (**C**,**D**) The taxa heatmap reveals the similarities and differences between the ALS and CON at the phylum level and the genus level (only top 20 displayed) on the basis of Bray–Curtis distance through 16S rDNA sequencing. The abundance profiles are transformed into Z scores by subtracting the average abundance and dividing the standard deviation of all samples, using signal sample (log2) to map the original value. The color gradient from blue to red is used to reflect the abundance from low to high. (**E**) The Cladogram results of LEfSe analysis show taxonomic clades that are differential in abundance, where different circles from the inside to the outside represent different classification levels from domain to species and the larger size of the nodes reflects higher relative abundance. The yellow nodes indicate no significant differences, and the biomarkers are colored in red and green depending on the group. (**F**) The bar chart shows the biomarkers with differential abundance between the groups and larger than the preset value (LDA score > 4, p < 0.05). The LDA score indicates the extent to which the corresponding group is affected by the differential microbes.

To further explore the characterization of the gut microbiota associated with ALS, we annotated the OTUs with both RDP and NT-16S database, generating numerous microbes at the level of domain, phylum, class, order, family, genus and species. The taxonomic distributions of fecal microbiota based on Bray–Curtis distance were shown in the stacked bar charts (Fig. 2A,B), where significant increases in the relative abundance of Bacteroidetes (42.23 vs. 26.46%, P = 0.0045) at the phylum level, as well as *Kineothrix* (0.0021 vs. 0%, P = 0.009), *Parabacteroides* (0.32 vs. 0.01%, P = 0.011), *Odoribacter* (2.61 vs. 1.87%, P = 0.0156), *Sporobacter* (0.12 vs. 0.02%, P = 0.0137), *Eisenbergiella* (0.21% vs. 0.07%, P = 0.0289), *Mannheimia* (0.12 vs. 0.02%, P = 0.0439), *Anaerotruncus* (0.03 vs. 0.02%, P = 0.0417) and *unclassified Porphyromonadaceae* (0.0097 vs. 0.0072%, P = 0.01) at the genus level, in addition to a significant reduction in Firmicutes (45.29 vs. 59.75%, P = 0.0248) at the phylum level and *Megamonas* (1.48 vs. 7.54%, P = 0.0335) at the genus level, were observed in the ALS group in comparison with the CON group. The chart of annotation at the genus level only exhibited the most abundant 20 microbes, which were therefore not completely in correspondence with the altered ones. Besides, the intestinal communities in rich abundance were as well depicted as the taxa heatmap (Fig. 2C,D). Moreover, according to LEfSe analysis (LDA score > 4, P < 0.05), Bacteria at the domain level (LDA = 4.89, p = 0.02), Bacteroidetes at the phylum level (LDA = 4.89, P = 0.02), Bacteroidia at the class level (LDA = 4.89, P = 0.02), Bacteroidales at the order level (LDA = 4.89, P = 0.02) and Porphyromonadaceae at the family level (LDA = 4.06, P = 0.002) were higher in ALS group, which could be deemed as potential candidate biomarkers to predict the risk of ALS (Fig. 2E,F). Taken together, these results suggested that the composition of gut microbiota was indeed affected in ALS patients.

### The prediction of Unigenes and functional annotation

**Figure 3:**
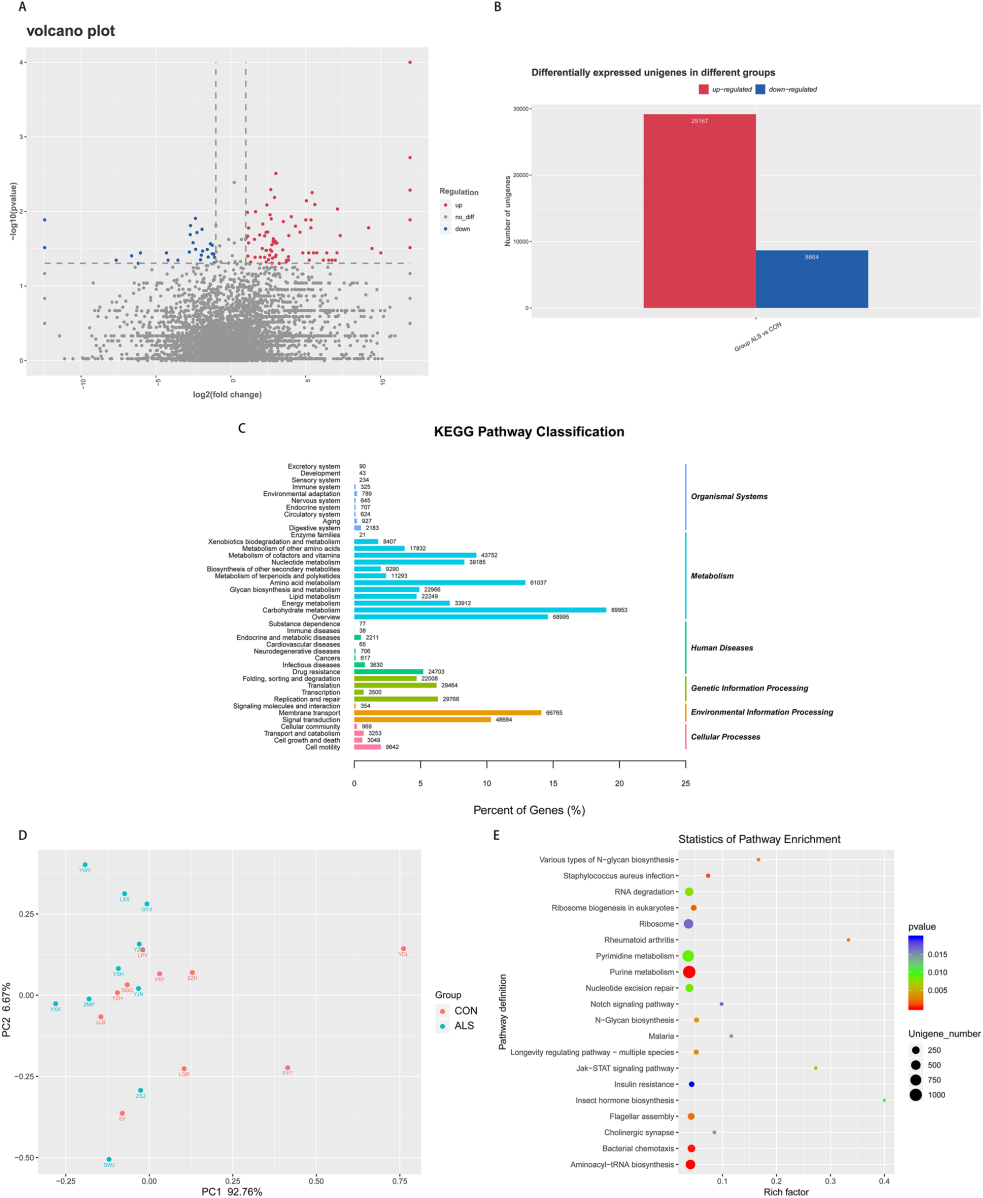
The expression pattern of Unigenes and gene functional annotation. (**A**) The volcano plot exhibits the overall distribution of Unigenes, where red dots represent the up-regulated ones, blue the down-regulated ones, and gray the ones that are not significantly different. (**B**) Statistical histogram of differentially expressed Unigenes. (**C**) Functional annotation of the whole Unigenes shown as 42 categories of KEGG pathways. The right and left vertical axis respectively refer to the primary and secondary classification information of KEGG pathway. (**D**) KEGG enrichment scatterplot in which 29 pathways are annotated with differentially expressed Unigenes. Rich factor measures the number of differential genes located in the specific KEGG, and larger rich factor indicates higher degree of enrichment. (**E**) PCA based on KEGG annotation reveals intra-group similarity and inter-group difference and shows that ALS group differs from CON group in the functional profiles. Each point represents a functional profile of the participants, which of the same group are presented in the same color. PC1 and PC2 refer to the principal components and the percentages shows the extent of variation explained. Dataset reference: Kanehisa, M. & Goto, S. KEGG: kyoto encyclopedia of genes and genomes. Nucleic Acids Res 28, 27–30, 10.1093/nar/28.1.27 (2000).

In order to reveal the potential distinction in gene function between ALS group and controls, the metagenomics was carried out. The results showed that 29,167 Unigenes were up-regulated, and 8,664 Unigenes were down-regulated in the ALS group compared to controls (Fig. 3A,B). Then the DIAMOND software was utilized to predict the gene function of the fecal microbiome via KEGG PATHWAY library and we annotated overall 42 different KEGG pathways (Fig. 3C). As for differentially expressed Unigenes, 29 significantly different KEGG pathways were identified in these two groups (Fig. 3D). The statistics of PCA were applied to reveal similarity within the group together with the difference between the group and as well indicated that the ALS group differed from controls in the functional profiles, where there was a significant decrease in pathways involved in metabolism of other amino acids (P = 0.0065), folding, sorting and degradation (P = 0.0082), nucleotide metabolism (P = 0.0413) and carbohydrate metabolism (P = 0.0494) in ALS group compared to the CON group (Fig. 3E). Thus, our data from the above exhibited the differentially expressed Unigenes and their function annotated using the KEGG databases, suggesting that the metabolic pattern in ALS patients might be distinctive from that in healthy controls.

### The conjoint analysis of metabolomics and metagenomics

**Figure 4:**
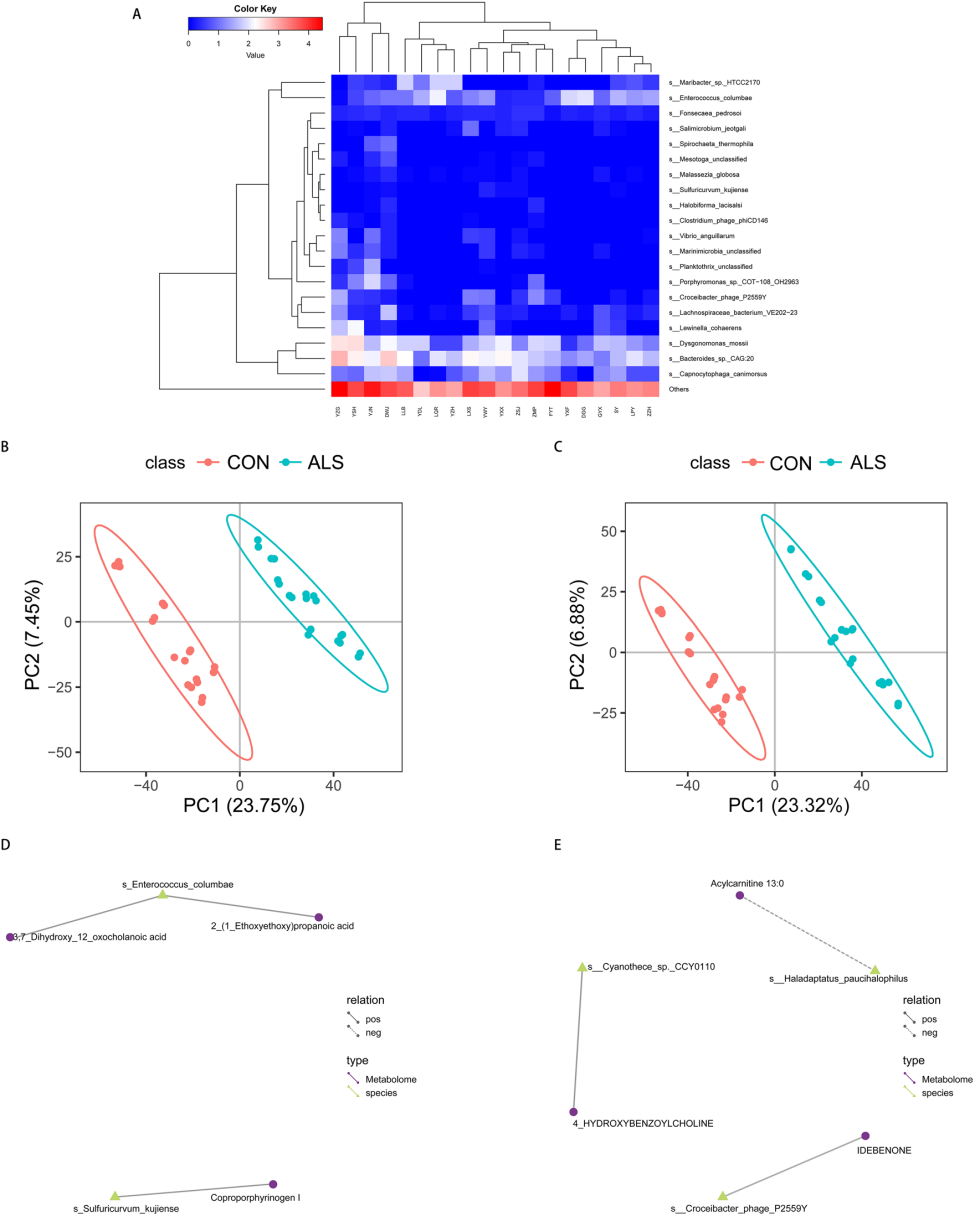
The associations of gut microbial metabolites and species. (**A**) Taxa heatmap via metagenomics reveals the microbial composition at the species level and only the top 20 in relative abundance based on Bray–Curtis distance are presented in the diagram. (**B**,**C**) PLS-DA score charts in the negative and positive ion mode are suggestive of the difference in metabolites between the groups (q ≤ 0.05, VIP ≥ 1.0 and |log2FC|> 1). The discreteness of symbols in different colors represent the distribution trend of the two groups on the PC1 and PC2 axes, respectively. (**D**,**E**) Network of differentially expressed species and metabolites in the negative and positive ion mode. The yellow triangles represent the species and the purple circles the metabolites. The solid line indicates a positive correlation, and the dashed line shows a negative correlation between the species and metabolites.

Taking the alteration in both microbial composition and the metabolic function into consideration, we then combined metagenomics with metabolomics to investigate the possible relationship between the metabolites and microbes. In comparison with controls, several microbes at the species level were significantly higher in the ALS group, including *Sulfuricurvum kujiense* (P = 0.006), *Cyanothece sp. CCY0110* (P = 0.0191) and *Haladaptatus paucihalophilus* (P = 0.0192), while *Enterococcus columbae* at the species level was significantly decreased (P = 0.0041), and the most relatively abundant 20 species based on Bray–Curtis distance were shown in the taxa map via metagenomics (Fig. 4A). Furthermore, PLS-DA was conducted to search for the differentially expressed metabolites between the two groups (q ≤ 0.05 ,VIP ≥ 1.0 and |log2FC|> 1). For metabolites detected in the negative ion mode (Fig. 4B,D), there was a positive correlation between *Enterococcus columbae* and 2-(1-ethoxyethoxy) propanoic acid (r = 0.85, p < 0.0001) as well as 3,7-dihydroxy-12-oxocholanoic acid (r = 0.84, p < 0.0001), and *Sulfuricurvum kujiense* was also positively correlated with coproporphyrinogen I (r = 0.82, p < 0.0001). As for those detected in the positive ion mode (Fig. 4C,E), we found that *Cyanothece sp. CCY0110* and 4-hydroxybenzoylcholine were positively correlated (r = 0.86, p < 0.0001), whereas *Haladaptatus paucihalophilus* and acylcarnitine 13:0 were negatively correlated (r = − 0.84, p < 0.0001).

In addition, our data suggested that the altered genes and pathways were correlated to metabolites regulation. On one hand, the KOEntry of *Enterococcus columbae* is K01809 (mannose-6-phosphate isomerase), which regulates carboxylic acids, steroids and their derivatives through the involvement in fructose and mannose metabolism as well as amino sugar and nucleotide sugar metabolism. On the other hand, K07407 (α-galactosidase) is the KOEntry of *Haladaptatus paucihalophilus* and regulates fatty acyls by contributing to galactose, glycerolipid and sphingolipid metabolism.

## Discussion

ALS is a neurodegenerative disease characterized by the presence of progressive degeneration of both upper motor neurons and lower motor neurons^26^. Heretofore, the pathogenesis of ALS remains not well understood^27^. Accumulating evidence suggests the multidirectional interaction between the central nerves system (CNS) and the gut microbiome which has been recently proven to have an impact on neuronal transmission, neuronal activity and complex host behaviors^28,29^.

In this study, we utilized 16S rDNA gene sequencing to figure out whether the constitution of stool microorganisms had a connection with ALS. After the high-throughput sequencing, we carried out quality control and pre-processing of the raw data, generating the clean data so as to ensure their reliability for the future analysis. In addition, the saturated rarefaction curve indicated that the sequences number of the samples was sufficient for the analysis. Though the result of PCoA was negative, the Shannon index of alpha diversity showed a significant difference in species richness between the ALS and controls. The Venn diagram and ANOSIM presented a different microbial composition in a more intuitionistic manner. The general microbial communities were relatively similar, but the specific OTUs were different between the ALS patients and healthy controls. Through the annotation of the OTUs, we found that the relative abundance of Bacteroidetes at phylum level and several bacteria at genus level in the ALS group were higher than those of the matched healthy individuals, while the former exhibited a lower ratio in Firmicutes at phylum level and *Megamonas* at genus level. LEfSe analysis further identified Bacteroidetes at the phylum level as one of the biomarkers for ALS, which was consistent with the aforementioned results. But this kind of difference at the phylum level may not be specific to ALS, since the vast majority of human gut intestinal microflora belongs to Firmicutes and Bacteroidetes phyla^30^. However, previous work has attested to the hypothesis that the Firmicutes to Bacteroidetes ratio was regarded as related to the susceptibility to disease status^31^. In this case, the higher richness of Bacteroidetes and lower abundance of Firmicutes, namely the decreased Firmicutes/Bacteroidetes ratio in ALS group potentially reflected the undermined health in patients with ALS. Therefore, the analysis indeed evinced that the community diversity and species constitution of fecal microbiota were in some way altered in ALS patients.

Since the amplicon sequencing purely involves single variable region (V4), the fecal microbiota could at most be determined at the genus level. To make a thorough inquiry into the differences between the ALS group and controls in the perspective of microflora gene function, shotgun sequencing was then applied, which can obtain more sequence information about microbes at the species level and is conducive to further gene annotation and pathway analysis from the molecular level. Based on the differentially expressed Unigenes, totally 29 categories of KEGG pathways were interpreted and the PCA demonstrated that several metabolic and intracellular processing pathways were declined in ALS patients. These results mirrored that apart from the imbalance in the quantity and composition of intestinal microbiota, the disturbed metabolic homeostasis thereof was as well associated with the predisposition to diseases. However, the differences in gene function annotated via KEGG library may not entirely in accord with the variations of microbial diversity. As indicated in a recent research, among uncorrelated individuals, the constitution of the gut microbiota is relatively specific while the function of it is more conservative^32^. Hence, it was more critical to take the metabolic effects into account than solely to concentrate on the taxon of gut microbial communities when committed to searching novel therapeutic targets.

To this end, metagenomics and metabolomics were combined to reveal the interaction between the gut microbiota and human hosts at a higher level. On one side, the application of shotgun sequencing detected several up-regulated fecal microbes and the down-regulated *Enterococcus columbae* at the species level. On the other side, in line with the correlation coefficients between the differential metabolites and species, we determined a few of pathways where both the microbiota and metabolites were involved. In reality, numerous studies have demonstrated that the intestinal microbial metabolites were implicated in the regulation of multiple physiological and pathological processes. It has been well known that short-chain fatty acids (SCFAs) produced by gut microbial fermentation modulated the absorption of a variety of nutrients and extensively participate in the energy metabolism^33^. As one of the main SCFAs in the intestine, butyrate was suggested to play a part in attenuating the abnormal accumulation of the mutated proteins in both ALS mice model and human intestinal epithelial cells^21^. With regard to metabolic diseases, microbial generation of imidazole propionate derived from histidine could disrupt insulin signaling and facilitate the development of insulin resistance, which eventually contributed to the onset of type II diabetes^34^. A recent study has also showed alteration in lipid metabolism including sphingolipids occurred prior to obvious neuropathy in an ALS mice model^35^. Thus, as presented in this study, the differentially expressed metabolites and microbiota could be deemed as potential dysbiosis biomarkers for ALS.

We noticed there are some inconsistencies between our data and a previous report which found insignificantly-altered diversity indices and taxa in the ALS patients compared to healthy controls^24^. Part of the discrepancy could be attributed to the methodological variation as we applied high-throughput sequencing technology. Another possibility is the racial heterogeneity for that the gut microbiome was relevant to the ethnicity^36^. Besides, a study analyzing and comparing the characteristics of ALS between the Chinese and German patients as well exhibited apparent differences in clinical phenotype, which further interpreted the contradiction^37^. The present research provides potential novel therapeutic targets for ALS exemplified by microbiota/metabolite transfer treatments. Nonetheless, we realized that our study still has certain limitations. As mentioned above, the gut microorganisms and relevant products are well involved in metabolic regulation and related to disease state. In this case, we could plausibly speculate that the disordered gut microbiota and metabolites acted through the gut–brain axis to play a role in the pathogenesis of ALS. Nevertheless, although we ruled out the possibility that the health conditions including immune and digestive functions of ALS patients affect the microbial constitution, our data simply suggested a correlation between the intestinal dysbacteriosis and ALS since we cannot exactly figure out whether the alteration of gut microbiota and metabolites is before or after the onset of ALS. Therefore, whether there is a pronounced causal relationship and how much this imbalance contributes to the etiology of ALS requires a further investigation under the framework of Koch’s postulates^38^. Furthermore, a multicenter and large-scale trial is as well needed to eliminate the unrelated differences among the subjects and to increase the reliability of the study.

To sum up, we probed into the discrepancies between the patients with probable or definite ALS and their caregivers who are healthy and close in lifestyle from the aspects of gut microbial community structure, gene function and the metabolites, which supported that the ALS might interact with the gut microbiota and established several potential biomarkers for further study of the occurrence and development of ALS. Up to now, a lot of scientists have devoted to developing the therapy that targets the pathogenesis of ALS and have achieved certain results. For instance, about 15% of cases can be attributed to dominant, high-penetrance gene variants, where gene editing is aimed at the inhibition of pathogenic gene toxicity and would perhaps serve as the treatment for hereditary ALS^39^. And intramuscular (IM) or intrathecal (IT) administration of autologous MSC-NTF cells (MSC: mesenchymal stem cell, NTF: neurotrophic factor) could postpone motor neuron degeneration and enhance motor performance^40^. In the present study, we highlight the role of intestinal flora in impacting ALS, and potential remedies may be achieved with cutting down the number of pathogens and promoting the growth of probiotics. Of note, our human data is observational and the number of subjects is relatively low, so that it is not sufficient to allow the specific treatment recommendation. However, our work to some extent lays a foundation for larger follow-up studies which might validate the causal effect of gut microbiome and even generate a new therapy.

## Methods

### Ethical statements and participants

The study was approved by the Ethical Review Board at the Xiangya Hospital of Central South University (No: 201703018). All participants had been given a written informed consent and all the methods were performed in conformity with the approved guidelines. We enrolled 20 eligible patients who were clinically diagnosed with probable or definite ALS according to the revised El Escorial criteria in neurology department of Xiangya hospital between October 2018 and February 2019^41^. Patients were excluded if they had cervical or lumbar spondylosis, multifocal motor neuropathy, Hirayama’s disease, spinal and bulbar muscular atrophy, syringomyelia, chronic Guillain–Barre syndrome or toxicosis. And we recruited 20 healthy matched controls living closely with the patient, to minimize the effects caused by different lifestyles and diets. All enrolled participants came from various cities in Hunan Province without racial difference and their living conditions and dietary structure were similar. Furthermore, exclusion criteria covered a wide range of gastrointestinal tract diseases and medications, history of gastrointestinal surgery, and a long-term history of obvious dietary nutritional imbalance that could respectively affect the intestinal microbiome.

### Specimen collection and storage

Fresh stool specimens were obtained from patients and the healthy individuals using special fecal collection device (provided by LC-Bio Technology Co. Ltd, Hang Zhou, Zhejiang Province, China). Afterwards, the researchers immediately collected two copies of fecal samples of about 2 g for each participant and placed them in the sterile centrifuge tube respectively. Then the centrifuge tubes with samples were frozen in liquid nitrogen for more than 4 h, followed by being transferred to − 80 °C fridge for preservation until final examinations.

### Sampling procedures and multivariate analysis

#### 16S rDNA sequencing

The total DNA was extracted using theStool DNA Kit (D4015-02, Omega, Inc., USA) according to manufacturer’s instructions, and then the V4 region of the prokaryotic (bacterial and archaeal) small-subunit (16S) rRNA gene was amplified with slightly modified versions of primers 515F (primer sequence: 5′- GTGYCAGCMGCCGCGGTAA-3′) and 806R (primer sequence: 5′- GGACTACHVGGGTWTCTAAT-3′) via polymerase chain reaction (PCR)^42^. The PCR products were confirmed with 2% agarose gel electrophoresis. Throughout the DNA extraction process, ultrapure water, instead of a sample solution, was used as a negative control to exclude the possibility of false-positive PCR results. The PCR products were purified by AMPure XT beads (Beckman Coulter Genomics, Danvers, MA, USA) and quantified by Qubit (Invitrogen, USA). Given that the sequencing flux of the early second-generation 454 platform is low and the sequencing cost is relatively high, it was sequenced on the MiSeq platform (Illumina) using the 2 $\times$ 250 bp paired-end protocol^43^.

Through the following chimeric sequences filtration and quality control, 1,735,678 total clean data were generated. After clustering the data into OTUs (operational taxonomic units) (at 97% identity) by VSearch software (v2.3.4)^44,45^, singletons and low-abundance ones (OTU abundance was less than 1/100,000 of the total sequence number of all samples) were filtered to reduce the false positive rate^46^. In total, 1,367 OTUs were available for further analysis.

Microbial diversity was measured by a range of OTU-based analyses of alpha- and beta-diversity calculated with QIIME software (Version 1.8.0)^47^. Alpha diversity was adopted in analyzing complexity of within-sample species diversity through 5 indices, including Chao1, Observed species, Goods coverage, Shannon and Simpson index. The unweighted and weighted UniFrac distances were applied to assess the beta diversity, which was used to evaluate differences between samples in species complexity. Venn diagram was portrayed to directly evaluate the common or specific OTUs between the samples.

Ribosomal Database Project (RDP) (https://rdp.cme.msu.edu/) and NT-16S database (ftp://ftp.ncbi.nlm.nih.gov/blast/db/FASTA/) were both employed for OTU annotation with BLAST program. The most abundant 20 communities were clustered on the basis of Bray–Curtis distance, and the relationship between species classification and sample distance was shown at the same time. LEfSe (Linear Discriminant Analysis Effect Size) was further analyzed to define the potential biomarkers with differences in abundance between the samples^48^.

### Metagenomics/Shotgun sequencing

To construct a metagenome library of 20 samples consisting of 10 ALS patients and 10 healthy controls, the shotgun sequencing was applied^49^. We first interrupted the genomic DNA using ultrasound, repaired the DNA fragment end, and added 'A' base to the 3′ end of DNA fragment. Then we added the sequenced junction, carried out fragment selection and PCR amplification, and finally checked the library and sequenced it on the computer to generate the raw data.

Low-quality data were filtered with the following pre-processing: (1) the splice sequence from the sequencing reads was removed; (2) the window quality scan, defined as 6 bp, of the sequenced reads was performed. The part from the beginning to the end of the window was truncated when the average quality value in the window was less than 20; (3) sequences with N content above 5% and length less than 60 bp after truncation were removed; (4) sequences with host contamination was removed. Then the filtered metagenomic reads were assembled by IDBA-UD program into contigs. Part of them with length larger than 500 bp were submitted to MetaGeneMark for Coding Region (CDS) prediction, followed by filtration of sequences whose CDS length is less than 100nt and de-redundancy with CD-HIT software. Afterwards, non-redundant genes were clustered with the identity of 95% and the coverage of 90%, and we selected the longest sequence as the representative. At last, we aligned all reads to genes with Bowtie2 and filtered out the genes where the number of reads was less than 2 in all samples to obtain final Unigenes.

The taxonomic analysis was performed with NCBI bacterial, archaeal, and viral non-redundant genome databases (ftp://ftp.ncbi.nlm.nih.gov/blast/db/FASTA/nr.gz). The DIAMOND software^50^ was employed to annotate the microbial gene function by comparing the protein sequences of Unigenes with those of KEGG (Kyoto Encyclopedia of Genes and Genomes) pathway library^51,52^.

### Metabolomics

The 40 collected samples were thawed on ice, and metabolites were extracted with 50% methanol buffer. The quality control samples were prepared at the same time (mixed with the same amount of prepared experimental samples). All chromatographic separations were performed using an ultra-performance liquid chromatography (UPLC) system (SCIEX, UK) with an ACQUITY UPLC T3 column (100 mm × 2.1 mm, 1.8 µm, Waters, UK). Then the metabolites eluted form the column were detected by a high-resolution tandem mass spectrometer TripleTOF5600plus (SCIEX, UK) which was operated in both positive and negative ion modes. The acquired data files were converted into mzXML format and then processed using XCMS^53^, CAMERA and metaX toolbox^54^ implemented with the R software. The KEGG database was used to annotate the metabolites by matching the exact molecular mass data (m/z) of samples with those from database.

### Statistical analysis

The statistical analysis of clinical baseline characteristics was performed via Stata (Version.11) and significantly difference was considered when the p-value was less than 0.05. The rest analysis was conducted using vegan package in R software. The Wilcoxon rank-sum test was used to compare the differences between the two groups of samples and the Spearman Correlation Coefficient was used to evaluate the degree of correlation between the two variables, where |r|> 0.8 indicated a significant correlation. The Principal Coordinates Analysis (PCoA) and Analysis of Similarities (ANOSIM) based on UniFrac distances were analyzed to evaluate the between-sample diversity in microbial structure. R value between − 1 and 1 was used as the statistic of ANOSIM, indicating the within-sample and between-sample differences. P < 0.05 and LDA (Linear Discriminant Analysis) score > 4 were set in the analysis of LEfSe. The Principal Component Analysis (PCA) was applied to reflect the differences in gene function on the basis of KEGG annotation. Rich factor reflected the number of differential genes located in the KEGG pathway. In 16S rDNA sequencing and metagenomics, differences were considered statistically significant when the p-value was less than 0.05. As to metabolomics, univariate analysis of fold change and t-test were used to correct the p-value via BH to obtain q-value, and the VIP (Variable Important for the Projection) value obtained by PLS-DA (Discriminant Analysis of Partial Least Square method) was used to screen differentially expressed metabolites (q ≤ 0.05, VIP ≥ 1.0, and |log2FC|> 1).

## Data Availability

Some or all data used to support the findings of this study are available from the corresponding author upon request.
